# Photosystem II supercomplexes lacking light‐harvesting antenna protein LHCB5 and their organization in the thylakoid membrane

**DOI:** 10.1111/ppl.70167

**Published:** 2025-03-24

**Authors:** Tereza Vánská, Roman Kouřil, Monika Opatíková, Iva Ilíková, Rameez Arshad, Pavel Roudnický, Petr Ilík

**Affiliations:** ^1^ Department of Biophysics Faculty of Science, Palacký University Olomouc Czech Republic; ^2^ Institute of Experimental Botany of the Czech Academy of Sciences, Centre of Plant Structural and Functional Genomics Olomouc Czech Republic; ^3^ Central European Institute of Technology, Masaryk University Brno Czech Republic

## Abstract

Light‐harvesting protein LHCB5 is one of the three minor antenna proteins (LHCB4‐6) that connect the core (C) of photosystem II (PSII) with strongly (S) and moderately (M) bound peripheral trimeric antennae (LHCIIs), forming a dimeric PSII supercomplex known as C_2_S_2_M_2_. Plants lacking LHCB4 and LHCB6 do not form C_2_S_2_M_2_, indicating that these minor antenna proteins are crucial for C_2_S_2_M_2_ assembly. However, studies on antisense *asLhcb5* plants suggest this may not apply to LHCB5. Using mild clear‐native PAGE (CN‐PAGE) and electron microscopy (EM), we separated and structurally characterized the C_2_S_2_M_2_ supercomplex from the Arabidopsis *lhcb5* mutant. When compared with wild type (WT), the C_2_S_2_M_2_ supercomplexes in the *lhcb5* mutant have slightly different positions of S and M trimers and are generally smaller and present in the thylakoid membrane at higher density. Using CN‐PAGE, we did not observe any PSII megacomplexes in the *lhcb5* mutant, although they are routinely detected by this method in WT. However, we identified the megacomplexes directly in thylakoid membranes via EM, indicating that the megacomplexes are formed but are too labile to be separated. While in WT, both parallel‐ and non‐parallel‐associated PSII supercomplexes can be detected in the thylakoid membrane (Nosek et al., 2017, *Plant Journal* 89, pp. 104–111), only the parallel‐associated PSII supercomplexes were found in the *lhcb5* mutant. This finding suggests that the formation of non‐parallel‐associated PSII supercomplexes depends on the presence of LHCB5. The presence of large PSII supercomplexes and megacomplexes, even though less stable, could explain the WT‐like photosynthetic characteristics of the *lhcb5* mutant.

## 1 INTRODUCTION

The primary reactions of oxygenic photosynthesis rely on the coordinated function of photosystems I (PSI) and II (PSII) in thylakoid membranes. These photosystems are composed of core complexes, varying numbers of light‐harvesting complexes (LHCs), and additional minor proteins, forming photosystem supercomplexes. These supercomplexes can associate into bigger megacomplexes and – in the case of PSII – into even larger structures known as semi‐crystalline arrays (Dekker & Boekema, 2005; Kouřil et al., 2018; Arshad et al., 2022). The photosystem assemblies, particularly those containing PSII, are highly variable and can adapt flexibly to environmental changes (e.g. Betterle et al., 2009; Kouřil et al., 2013). Still, the physiological significance of PSII assemblies remains poorly understood. The PSII supercomplex typically consists of a core dimer (C_2_) with attached LHCs. In land plants, the main peripheral PSII antenna is composed of two strongly (S_2_, formed by LHCB1 and LHCB2) and two moderately (M_2_, formed by LHCB1‐3) bound light‐harvesting trimers (LHCIIs). The trimeric LHCIIs are connected to the PSII core by minor monomeric antennae LHCB4‐6 (also named CP29, CP26, and CP24, respectively), forming stable supercomplex C_2_S_2_M_2_ (Caffarri et al., 2009; Albanese et al., 2016; van Bezouwen et al., 2017; Su et al., 2017). Minor antennae LHCB4 and LHCB5 mediate the binding of the S LHCII trimer, while LHCB4 and LHCB6 antennae are important for the attachment of the M LHCII trimer (Ballottari et al., 2007; Caffarri et al., 2009; Kouřil et al., 2013; Kim et al., 2020). The only exception is the Pinaceae family, which has retained a single isoform of LHCB4, called LHCB8 (Grebe et al., 2019; Opatíková et al., 2023), while losing LHCB6 and LHCB3, and where the M trimer appears to be attached to the core in a different manner not observed in other land plants (Kouřil et al., 2016, 2020; Ilíková et al., 2021).

Among the minor light‐harvesting antennae, LHCB5 is the most similar to the monomers that constitute trimeric LHCIIs and shares several features with them, such as spectral properties and structural and pigment composition (Croce et al., 2002; Marin et al., 2010, 2011). Although LHCB5 is typically present as a monomer, under specific conditions, it was reported to be able to form trimers similar to those formed by LHCB1‐3 (Ruban et al., 2003). However, the ability of LHCB5 to form trimers has recently been questioned and requires a more detailed investigation (Sattari Vayghan et al., 2022).

The importance of the individual minor antennae LHCB4‐6 for the assembly of PSII supercomplexes and megacomplexes has been studied using *Arabidopsis thaliana* (Arabidopsis) mutants. Among the three monomeric antennae, the absence of LHCB4 appears to have the most profound effect on the stability of the PSII supercomplex. In the *lhcb4* mutant, the presence of a small amount of C_2_S_2_M_(2)_ was only inferred, but this type of supercomplex has not yet been successfully separated (De Bianchi et al., 2011). The overall instability of PSII supercomplexes is probably the reason why larger assemblies like semi‐crystalline arrays are completely absent in thylakoid membranes of the *lhcb4* mutant (De Bianchi et al., 2011; Goral et al., 2012). In the absence of LHCB6, the largest form of PSII supercomplex found in Arabidopsis is C_2_S_2_. However, these C_2_S_2_ supercomplexes are much more stable than in the *lhcb4* mutant and form semi‐crystalline arrays in thylakoid membranes (Kovács et al., 2006). The arrays are even more extensive in the double mutant lacking both LHCB6 and LHCB3, where virtually all C_2_S_2_ supercomplexes are arranged into arrays (Ilíková et al., 2021).

Unlike LHCB4 and LHCB6, the role of the third minor antenna, LHCB5, in the assembly of C_2_S_2_M_2_ supercomplexes seems to be not essential. In the antisense *aslhcb5* plants, Yakushevska et al. (2003) found semi‐crystalline PSII arrays in EM micrographs, which they successfully fitted using WT‐like C_2_S_2_M_2_ without LHCB5. However, the structural details of this supercomplex could not be determined by this approach. Their attempt to separate large native PSII supercomplexes from *aslhcb5* by gel‐filtration chromatography resulted in the retrieval of only C_2_S_2_ supercomplexes, implying that C_2_S_2_M_2_ without LHCB5 are very unstable. Similarly, the separation of supercomplexes from *lhcb5* insertion mutant by sucrose gradient centrifugation also yielded only C_2_S_2_ as the largest PSII supercomplex form (Caffarri et al. 2009). Recently, Chen et al. (2018) separated PSII supercomplexes from the T‐DNA insertion *lhcb5* plants using blue‐native PAGE and obtained the band pattern attributable to PSII supercomplexes similar to WT. However, in this paper, the separated PSII supercomplexes were not structurally identified, and the authors used plants grown in uncontrolled field conditions, including high light priming.

Based on the available data, it seems that the C_2_S_2_M_2_ PSII supercomplexes can be formed in plants lacking LHCB5, but these supercomplexes have not been separated, identified and structurally characterized. In our study, we have successfully separated PSII supercomplexes from the *lhcb5* Arabidopsis mutant grown under normal controlled conditions using very mild CN‐PAGE and characterized them by single‐particle EM. This approach allowed us to separate even the largest form of PSII supercomplex ‐ C_2_S_2_M_2_. Due to the absence of the LHCB5 antenna, the S and M trimers were slightly shifted compared to C_2_S_2_M_2_ from WT. The analysis of EM images of PSII particles in grana membranes of the *lhcb5* mutant showed that LHCB5‐less PSII supercomplexes are able to form specific PSII megacomplexes, consisting of two parallel‐associated PSII supercomplexes, as well as PSII semi‐crystalline arrays.

## 2 MATERIALS AND METHODS

### 2.1 Plant material

*Arabidopsis thaliana* wild type (WT, accession Columbia) and T‐DNA insertion mutant line *lhcb5* (SALK_014869c) were obtained from the Nottingham Arabidopsis Stock Centre (NASC) collection. Arabidopsis plants were grown in soil in a Phytoscope walk‐in chamber (Photon Systems Instruments, Drásov, Czech Republic) in controlled conditions: 8 h light/16 h dark cycle; 22/20°C; 110 μmol photons m^−2^ s^−1^; 60% humidity. Six to seven‐week‐old plants were used for experiments.

### 2.2 *In vivo* measurements

Analysis of the samples was performed as described previously (Ilíková et al., 2021). The plants used for the measurements were taken from the middle of the light period and dark‐adapted for 30 min. All *in vivo* chlorophyll fluorescence measurements were performed in dim green light. The fast chlorophyll fluorescence induction curve was measured using a Plant Efficiency Analyser (PEA, Hansatech). Leaves were exposed to red actinic light (4 000 μmol photons m^−2^ s^−1^) for 10 s. Minimal (F_O_) and maximal (F_M_) chlorophyll fluorescence levels and maximum quantum yield of PSII photochemistry F_V_/F_M_ (F_V_ = F_M_ – F_O_) were evaluated using the Biolyzer software (R.M. Rodriguez, University of Geneva).

A Dual‐PAM100 measuring system (Heinz Walz) was used to estimate the PSI and PSII performance simultaneously. Leaves of dark‐adapted Arabidopsis plants were exposed to red actinic light for 16 min (800 μmol photons m^−2^ s^−1^) with 300 ms saturating pulses (10 000 μmol photons m^−2^ s^−1^). The effective PSII quantum yield was calculated according to Genty et al. (1989) as Y(II) = (F_M_^’^ – F)/F_M_^’^, where F_M_^’^ represents the maximum chlorophyll fluorescence of an illuminated sample and F is chlorophyll fluorescence level at the state induced by the actinic light. Moreover, non‐photochemical quenching parameter (NPQ) was calculated as (F_M_ – F_M_^’^)/F_M_^’^, where F_M_ represents maximum fluorescence in a dark‐adapted state. The effective quantum yield of PSI photochemistry Y(I) was estimated during chlorophyll fluorescence induction using simultaneous measurement of absorption changes related to redox changes of the primary donor of PSI (P700) according to Klughammer and Schreiber (2008) using the Dual‐PAM100 software.

### 2.3 Fresh weight determination and isolation of thylakoid membranes

Plants were dark‐adapted for 30 min before isolation of thylakoid membranes. Arabidopsis rosettes were cut at the base for the determination of fresh weight and subsequently, intact thylakoid membranes were isolated according to Dau et al. (1995) with modifications. Rosettes were homogenized in grinding buffer (35 mM HEPES/NaOH, pH 7.2, 0.4 M NaCl, 0.4 M sucrose, 4 mM MgCl_2_·6H_2_O, 5 mM sodium ascorbate, 2 mg ml^−1^ BSA). The homogenate was filtered through two layers of Miracloth and centrifuged (5000 *g*, 4°C, 6 min). Pellet was resuspended in buffer (25 mM HEPES/NaOH, pH 7.5, 150 mM NaCl, 8 mM MgCl_2_·6H_2_O, 0.9 mM sodium‐EDTA) and centrifuged (5000 *g*, 4°C, 10 min). The pellet was resuspended in stock buffer (50 mM HEPES/NaOH, pH 7.2, 0.4 M sucrose, 15 mM NaCl, 5 mM MgCl_2_·6H_2_O) and centrifuged (5000 *g*, 4°C, 5 min). Finally, the pellet containing intact thylakoids was resuspended in a small amount of the stock buffer. All procedures were performed under green light on ice or at 4°C. To estimate the chlorophyll content of the isolated thylakoid membranes, pigments were extracted into 80% acetone and determined spectrophotometrically according to Lichtenthaler (1987).

### 2.4 Pigment analysis

Leaves for pigment analysis were collected from dark‐adapted plants (30 min) while the area and the weight of individual leaves were determined. Leaves were shock‐frozen in liquid nitrogen and stored at −80°C. The frozen leaves were grinded in a mortar with a pinch of MgCO_3_ in 80% cold acetone. The homogenates were centrifuged (5000 *g*, 4°C, 10 min) and pigment content was estimated in supernatants. The contents of chlorophyll a, chlorophyll b and the sum of carotenoids were estimated spectrophotometrically according to Lichtenthaler (1987).

### 2.5 CN‐PAGE

CN‐PAGE was performed according to Nosek et al. (2017) with minor modifications. Thylakoid membranes (10 μg of chlorophyll) were solubilized with n‐dodecyl‐α‐D‐maltopyranoside (α‐DDM) using a detergent:chlorophyll mass ratio of 10, and supplemented with sample buffer (50 mM HEPES/NaOH, pH 7.2, 0.4 M sucrose, 5 mM MgCl_2_, 15 mM NaCl, 10% glycerol) to a final volume of 30 μL. Samples were gently mixed, incubated (1 min, 4°C), and centrifuged (20 000 *g*, 4°C, 10 min) and supernatants were loaded into wells of 4–8% polyacrylamide gradient gels (Wittig et al., 2007). The electrophoretic separation was conducted using Bio‐Rad Mini‐PROTEAN Tetra cell system with bis‐tris/tricine buffer system (cathode buffer – 50 mM tricine, 15 mM bis‐tris/HCl, pH 7.0; anode buffer ‐ 50 mM bis‐tris/HCl, pH 7.0) in the dark with ice cooling. Gel was run with an initial constant current of 3.5 mA for 15 min, then the current was increased to 7 mA. The gel was analyzed using a gel scanner Amersham Imager 600RGB (GE HealthCare Life Sciences, Tokyo, Japan) in transmission mode using white light illumination.

In order to compare the abundances of PSII supercomplexes in the gel, densitometric analysis was performed using the public ImageJ software (ImageJ 1.53 t, National Institutes of Health, Bethesda, MD, USA; Schindelin et al., 2012). Areas under the peaks in the densitograms representing the individual PSII supercomplexes were determined. Ratios of densities for corresponding PSII supercomplexes (i.e. C_2_S_2_M_2_, C_2_S_2_M and C_2_S_2_/C_2_SM bands) in *lhcb5* and WT were calculated.

### 2.6 Electron microscopy

Specimens for transmission electron microscopy were prepared on glow‐discharged carbon‐coated copper grids and negatively stained with 2% uranyl acetate. Electron micrographs were collected using a Tecnai G2 F20 microscope (FEI Technologies, Hillsboro, USA) with an Eagle 4 K CCD camera (FEI Technologies, Hillsboro, USA).

Single particle electron microscopy of different forms of PSII supercomplexes from Arabidopsis *lhcb5* mutant was performed after their extraction from the gel. Two to three bands were cut out from the gel for each sample. The bands were cut into small pieces and placed into elution buffer (50 mM HEPES, 15 mM NaCl, 5 mM MgCl_2_, α‐DDM at a critical micellar concentration of the detergent – 0,008%, pH 7.2) and eluted in the fridge overnight. Obtained supernatants were directly used for specimen preparation. Electron micrographs were recorded at 134 028x magnification. The pixel size at the specimen level after binning the images to 2 048 x 2 048 pixels was 0.224 nm. Approximately 6 000, 24 000, and 23 000 PSII particle projections were picked in semi‐automated mode from 10 092, 8 524, and 1 725 micrographs of specimens prepared from the gel bands assigned as C_2_S_2_M_2_, C_2_S_2_M, and C_2_SM, respectively. Individual datasets were subjected to reference free two‐dimensional classification using the SCIPION image processing framework (De la Rosa‐Trevín et al., 2016). The structure of the C_2_S_2_M_2_ supercomplex (Van Bezouwen et al., 2017) was modified and used to fit the projection maps of analyzed *lhcb5* PSII supercomplexes.

Electron micrographs of grana membranes from *A. thaliana* WT (185 micrographs) and *lhcb5* mutant (268 micrographs), isolated according to Kouřil et al. (2013), were recorded at 83 562x magnification. The pixel size at the specimen level after binning the images to 2 048 x 2 048 pixels was 0.36 nm.

In the case of the analysis of specific PSII megacomplexes in the *lhcb5* mutant, about 52 000 projections of PSII particles were selected from the grana membranes and analyzed. In the case of the analysis of two‐dimensional crystalline arrays of PSII supercomplexes from *lhcb5* mutant, selected sub‐areas (1080 x 1080 Å), lattice parameters of the crystalline arrays and a ratio of the area of semi‐crystalline PSII arrays per total area of the grana membranes were analyzed as in Ilíková et al. (2021).

To determine the minimal distances between PSII complexes, approximately 37 000 and 30 000 PSII projections were selected using SCIPION from micrographs of grana membranes without PSII arrays isolated from *A. thaliana* WT and *lhcb5* mutant. The coordinates of the PSII particles were then exported and analyzed using Microsoft Excel.

### 2.7 Western‐blotting

Analysis of the samples was performed as described previously (Ilíková et al., 2021). Proteins for immunoblot analysis were isolated from thylakoid membrane suspensions with extraction buffer (14 mM DL‐dithiothreitol, 28 mM Na_2_CO_3_, 175 mM sucrose, 5% (w/v) SDS, and 10 mM EDTA‐Na_2_). Thylakoid suspension (100 μg of chlorophylls) was mixed with 1 mL of the extraction buffer and incubated for 30 min at 70°C. After centrifugation (10 min, 19 200 *g*), the supernatants were used for blotting. The volume of isolated proteins corresponding to 0.5 or 1 μg of chlorophylls was used together with sample buffer (Tricine Sample Buffer, BioRad; 3x diluted) and dH_2_O. The sample mixture was then incubated for 10 min at 70°C, and samples were loaded onto 10% gel (Mini‐PROTEAN TGX Precast Protein Gel, Bio‐Rad) with tris/tricine buffer system (Schägger, 2006). Electrophoretic separation voltage was constant (100 V) for 45 min. Proteins from the gel were transferred onto a polyvinylidene fluoride membrane using Trans‐Blot Turbo RTA Mini 0.2 μm PVDF Transfer Kit (BioRad). LHCB5 protein was detected using Agrisera antibody. Primary antibody Anti‐LHCB5 (AS01 009; dilution 1:5000) was used for the detection of the LHCB5 protein in samples, the presence of the primary antibody was detected using a secondary antibody with conjugated HRP enzyme. Visualization of the chemiluminescent signal was achieved by developing with Immobilon Western Chemiluminescent HRP Substrate (Merc) with signal capturing using gel scanner Amersham Imager 600 RGB (GE HealthCare Life Sciences).

### 2.8 Mass spectrometry

Samples of thylakoid membranes were divided into three technical replicates, which were separately subjected to filter‐aided sample preparation as described elsewhere (Wiśniewski et al., 2009). The resulting peptides from all samples were analyzed by liquid chromatography–tandem mass spectrometry (LC–MS/MS) performed using UltiMate 3000 RSLCnano system (Thermo Fisher Scientific) online coupled with timsTOF Pro mass spectrometer (Bruker). See the supporting information section for full details regarding the analyses and data evaluation.

## 3 RESULTS AND DISCUSSION

### 3.1 Phenotypic characterization of the *lhcb5* mutant

**FIGURE 1:**
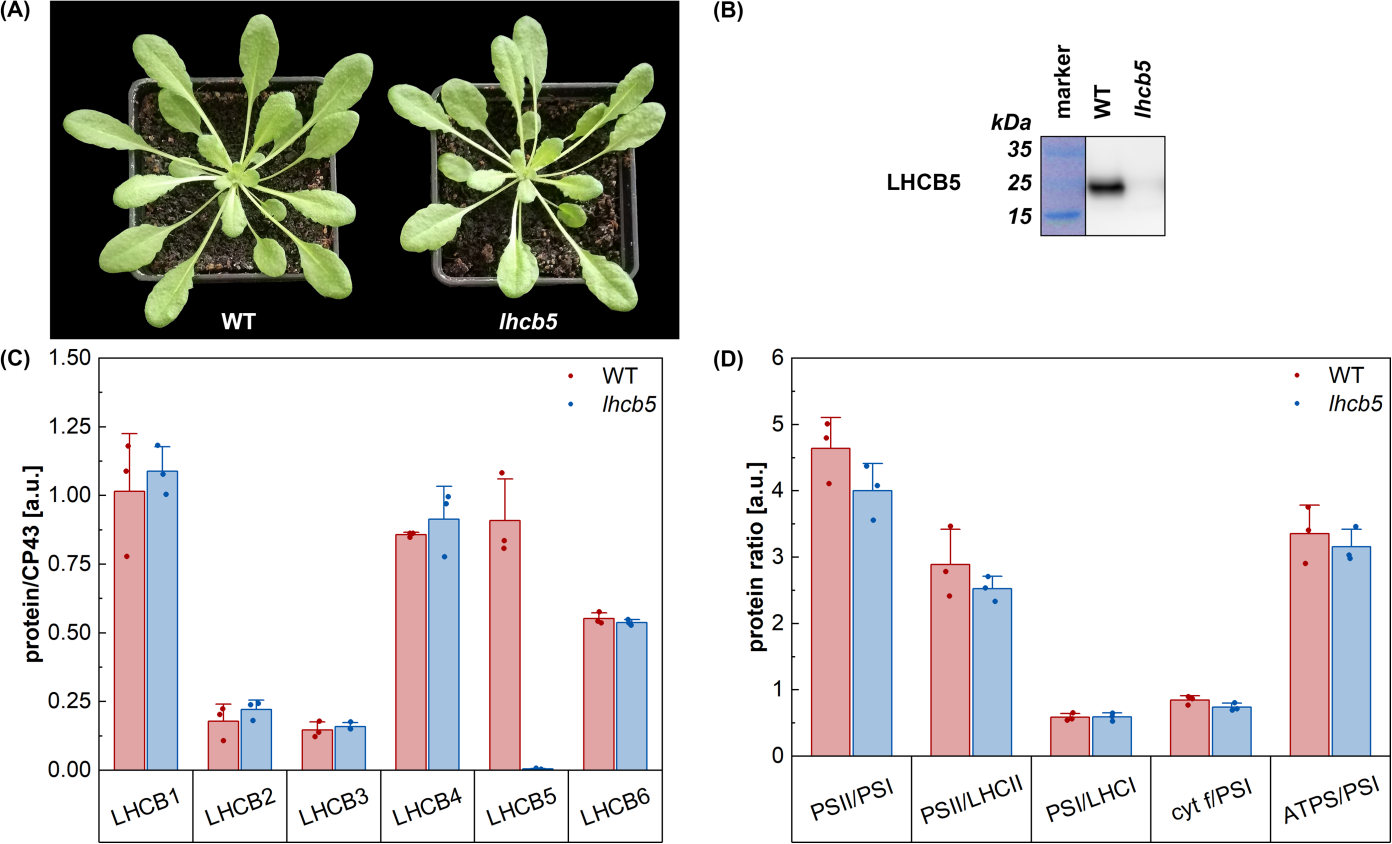
**Phenotype and photosynthetic characteristics of the lhcb5 mutant**. (A) Phenotype of *Arabidopsis thaliana* wild type (WT) and *lhcb5* mutant plants grown at controlled conditions for 6 weeks (8 h light/16 h dark cycle; 22/20°C; 110 μmol photons m^−2^ s^−1^; 60% humidity). (B) Immunoblot analysis of thylakoid membranes of WT and *lhcb5* mutant plants with antibody directed against LHCB5. (C) Content of light‐harvesting proteins LHCB1‐6 evaluated relatively to the content of CP43 protein in the WT and the *lhcb5* mutant. The protein content was determined in isolated thylakoid membranes by liquid chromatography–tandem mass spectrometry (LC–MS/MS). The columns represent means + SD, individual points show technical replicates. All data passed the normality and equal variance tests and according to Student t‐test the datasets of WT and *lhcb5* were not significantly different (α ≤ 0,05), except for the relative content of LHCB5/CP43. (D) Protein ratios of photosynthesis‐related thylakoid membrane proteins of the WT and the *lhcb5* mutant. The protein content was determined by LC–MS/MS in isolated thylakoid membranes. PSII represents the sum of relative PG intensities of D1, D2, CP43, and CP47 proteins, LHCII ‐ LHCB1–3 proteins, PSI ‐ PSAA and PSAB proteins, LHCI ‐ LHCA1–4 proteins, ATPS ‐ α and β subunits of ATP synthase, and cyt f represents cytochrome f component of cytochrome b_6_f complex. The columns represent means + SD, individual points show technical replicates. All data passed the normality and equal variance tests and according to Student t‐test the datasets of WT and *lhcb5* are not significantly different (α ≤ 0,05).

Previous studies on plants lacking LHCB5 suggest that the absence of this minor antenna does not have any profound effect on plant phenotype (De Bianchi et al., 2008; Chen et al., 2018). This was confirmed by our data, as also under our growth conditions the *lhcb5* mutant did not show visible phenotypic change compared to WT (Figure 1A, Table S1). The absence of LHCB5 protein in the mutant was confirmed by immunoblot analysis (Figure 1B) and proteomic analysis showed that the loss of LHCB5 affects neither the relative ratios of the major photosynthetic complexes (PSI, PSII, LHCII, cytochrome b_6_/f complex and ATP synthase; Figure 1D), nor the relative amount of remaining PSII light‐harvesting proteins (Figure 1C). The latter result, which implies that the missing LHCB5 antenna in the mutant was not replaced by any other LHCB antenna protein, is consistent with the data obtained by Chen et al. (2018) on *lhcb5* plants grown in field conditions. Interestingly, a previous study by De Bianchi et al. (2008) suggested that the absence of LHCB5 might be partially compensated by LHCB4 and LHCB6, as both minor antennae appeared to be upregulated on Western blots. In the aforementioned study, however, the plants were grown in conditions with unusually high humidity (90%), which may have affected their physiology.

Data from the literature indicate that the photosynthetic parameters of the *lhcb5* mutant are very similar to WT (De Bianchi et al., 2008; Betterle et al., 2009; Van Oort et al., 2010; Miloslavina et al., 2011; Chen et al., 2018). However, as different growth regimes can affect plant performance, we performed basic chlorophyll fluorescence measurements on *lhcb5* mutants to verify the finding under our conditions. The photochemical yield of both photosystems during the dark‐to‐light and light‐to‐dark transitions, as well as NPQ induction, were not affected by the absence of LHCB5 (Figure S1). The presence of weakly bound LHCII trimers (typical for *lhcb6* mutant) is known to lead to increased F_O_ parameters (Ware et al., 2015; Ilíková et al., 2021). No such change was observed in the *lhcb5* mutant (Table S1), which indicates that LHCII antennae are well connected to the reaction center even in the absence of LHCB5 in dark‐adapted plants. The same conclusion for dark‐adapted *lhcb5* was reached by Miloslavina et al. (2011) based on the measurement of chlorophyll fluorescence decay kinetics. On the other hand, the maximal chlorophyll fluorescence level (F_M_) was slightly reduced in the *lhcb5* mutant compared to WT, which reflects some modification in PSII function at saturation light intensity. The decrease in F_M_ level slightly reduces the maximum quantum yield of PSII photochemistry detected as the F_V_/F_M_ ratio (Table S1).

### 3.2 Characterization of PSII supercomplexes in the *lhcb5* mutant

We expected that PSII supercomplexes in *lhcb5* mutant plants grown under standard controlled conditions are very labile (see Introduction), thus we aimed to separate native PSII supercomplexes from thylakoid membranes of *lhcb5* plants using the approach we have already successfully used in our previous study on Arabidopsis mutants (Ilíková et al., 2021).

**FIGURE 2:**
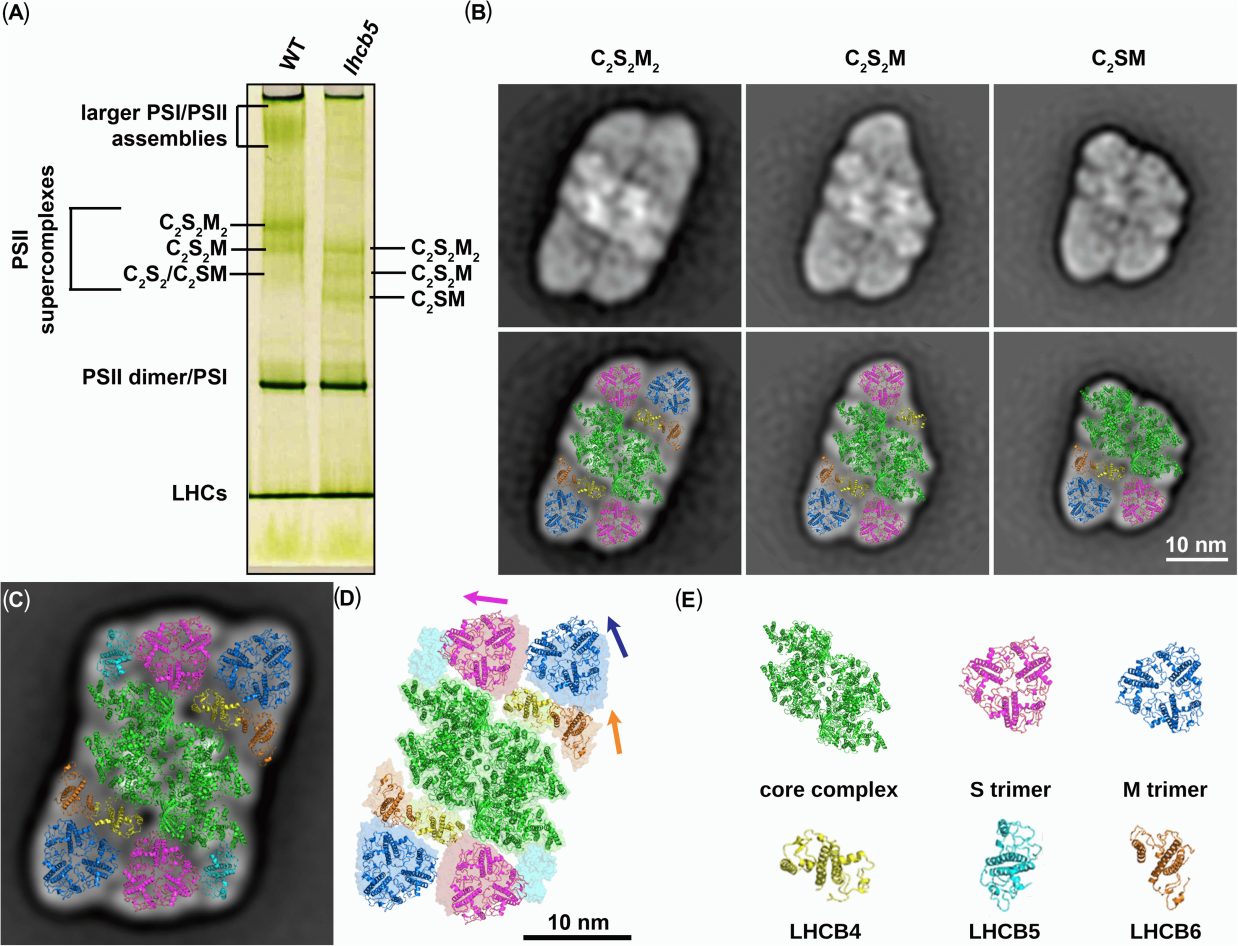
**Separation and structural characterization of PSII supercomplexes from lhcb5 mutant plants**. (A) Separation of pigment–protein complexes from thylakoid membranes from *Arabidopsis thaliana* WT and *lhcb5* mutant plants by clear native polyacrylamide gel electrophoresis. Thylakoid membranes were solubilized by n‐dodecyl α‐D‐maltopyranoside (detergent/chlorophyll mass ratio of 10). (B) Electron density maps of characteristic PSII supercomplexes from the separated green gel bands of the *lhcb5* mutant designated as C_2_S_2_M_2_, C_2_S_2_M and C_2_SM. Projection maps are fitted by corresponding structural high‐resolution models of PSII supercomplexes (Van Bezouwen et al., 2017) without LHCB5. Individual PSII subunits are color‐coded according to (E). (C), (D) Comparison of structural models of the PSII C_2_S_2_M_2_ supercomplexes from *Arabidopsis thaliana* WT and the *lhcb5* mutant. (C) Projection map of the PSII C_2_S_2_M_2_ supercomplex from *Arabidopsis thaliana* wild type (Ilíková et al., 2021) fitted by the high‐resolution structure from Van Bezouwen et al. (2017). (D) Overlay of structural models of the PSII C_2_S_2_M_2_ supercomplex from *Arabidopsis thaliana* wild type (surface representation, partially transparent) and the *lhcb5* mutant shows a specific shift of the S and M LHCII trimers as well as the monomeric antenna LHCB6 (see arrows in the corresponding colors) due to the absence of LHCB5. Individual PSII subunits are color‐coded according to (E). (E) Legend of individual PSII subunits, which are color‐coded as follows: PSII core complex in green, S and M LHCII trimers in magenta and blue, respectively, and the monomeric antenna proteins, LHCB4, LHCB5, LHCB6, in yellow, cyan, and dark orange, respectively.

The protocol, based on the solubilization of thylakoid membranes with α‐DDM followed by mild CN‐PAGE, enables the separation of large amounts of C_2_S_2_M_2_ supercomplexes and PSII megacomplexes from WT Arabidopsis (Nosek et al., 2017). Indeed, the analysis of WT thylakoid membranes resulted in the expected green band pattern typical for WT (Figure 2A; Nosek et al., 2017; Kouřil et al., 2018; Ilíková et al., 2021). In the case of the *lhcb5* mutant, we have observed a similar fingerprint of the PSII supercomplex bands (Figure 2A). All the bands were shifted on the gel compared to their WT counterparts, which is expected, as the supercomplexes lack the LHCB5 minor antenna.

Particles from all three *lhcb5* supercomplex bands were analyzed in detail using single‐particle EM analysis. The most abundant protein complex in the uppermost band was PSII supercomplex C_2_S_2_M_2_ (Figure S2), which clearly lacks the LHCB5 antenna (Figure 2A, B). Comparison of the models of C_2_S_2_M_2_ supercomplex from WT (Figure 2C) and *lhcb5* (Figure 2B) showed that the missing LHCB5 protein leads to the shift of M and S trimers as well as LHCB6 antenna towards the empty binding site of LHCB5 (Figure 2D). The middle CN‐PAGE band contains, along with the intact C_2_S_2_M supercomplex, a notable amount of C_2_S_2_M_2_ particles and smaller particles, which represent contamination from the surrounding bands and breakdown products formed during sample preparation for EM (Figure S3). The lowest band contains C_2_SM supercomplex as the most abundant form (Figure S4). Interestingly, this band does not contain C_2_S_2_ supercomplexes, which in WT usually co‐migrate with C_2_SM supercomplexes (Caffarri et al., 2009). This finding can be interpreted as a strong indication that the absence of LHCB5 weakens the binding of the S trimer to the dimer core, leading to destabilization of the entire supercomplex. The abundance of PSII supercomplexes was determined by densitometric analysis of the gels (Table S2). The densities of the bands corresponding to larger supercomplexes C_2_S_2_M_2_ and C_2_S_2_M were reduced in the *lhcb5* mutant compared to WT by about 40% and 26%, respectively. Conversely, the band corresponding to the C_2_S_2_/C_2_SM supercomplex was denser by about 35% in the *lhcb5* mutant. These findings evidence the reduced stability of the larger PSII supercomplexes in the absence of the LHCB5 minor antenna, as was also documented in previous studies (Yakushevska et al., 2003; Caffarri et al., 2009). On the other hand, conformational changes of the PSII supercomplexes associated with the absence of LHCB5 may alter the accessibility of phosphorylation sites of various PSII proteins, which could be particularly important, e.g. under field stress conditions. This, in turn, may contribute to the increased stability of these PSII supercomplexes (see Chen et al., 2018).

The wide band visible in the upper part of the CN‐PAGE gel of WT thylakoid membranes (Figure 2A) is ascribed to larger PSI/PSII assemblies, including megacomplexes (Caffarri et al., 2009; Nosek et al., 2017; Ilíková et al., 2021). This band is completely missing in the electrophoretogram of the *lhcb5* mutant (Figure 2A), indicating either very low stability or a complete absence of megacomplexes. The important role of LHCB5 in the assembly of the PSII megacomplexes has already been hypothesized previously (Nosek et al., 2017).

### 3.3 Organization of PSII supercomplexes on the membrane level

**FIGURE 3:**
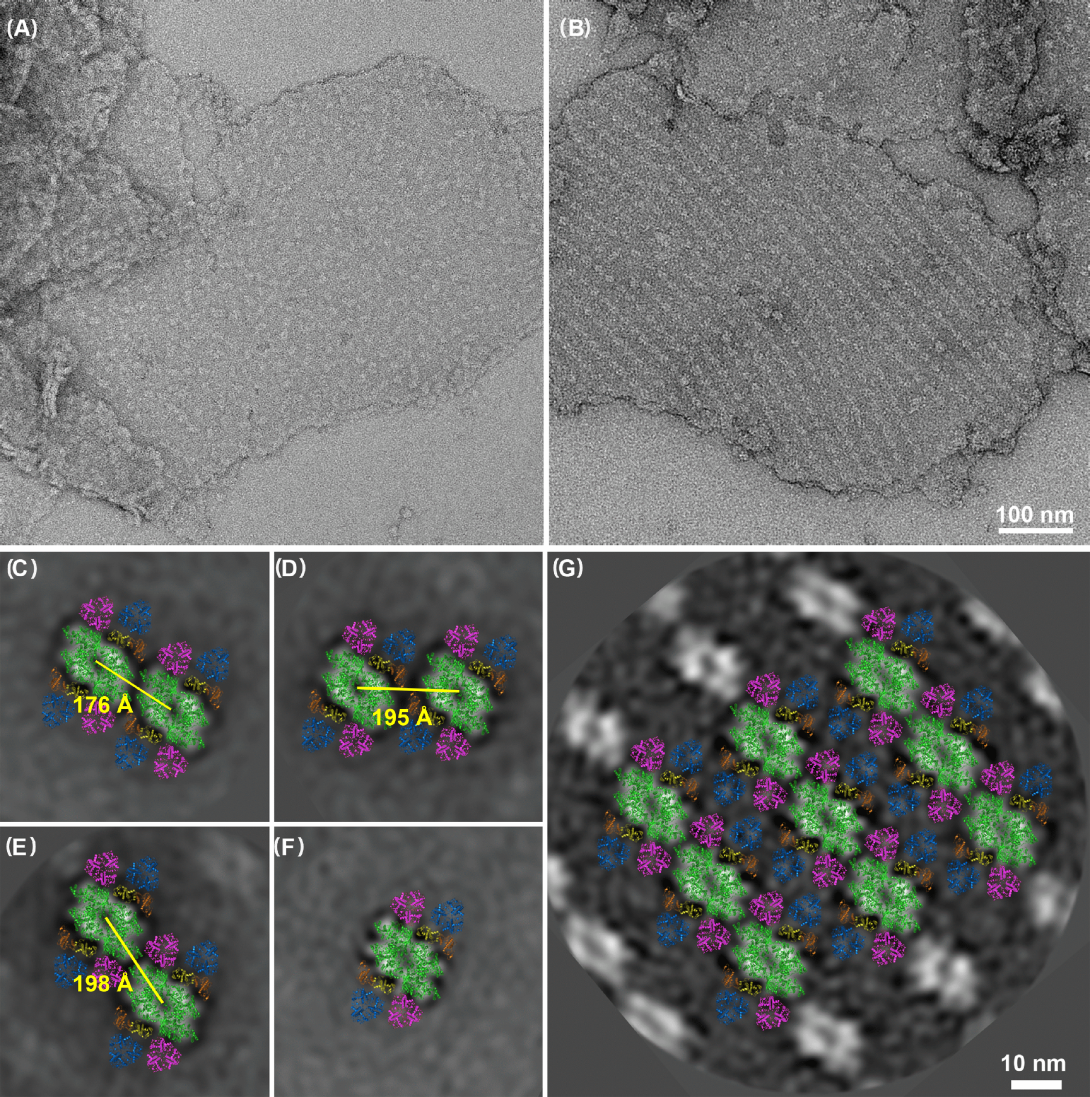
**Organization of photosystem II in thylakoid membranes of the lhcb5 mutant**. (A, B) Examples of electron micrographs of negatively stained thylakoid membrane isolated from the *lhcb5* mutant with densities corresponding to the PSII core complex. Representative picture of PSII supercomplexes “randomly” organized (A) and organized into 2D semi‐crystalline array (B). (C, D, E) Projection maps of PSII megacomplexes obtained using image analysis of PSII particles in thylakoid membranes. Three specific associations of PSII supercomplexes are shown and fitted by the model of PSII supercomplex C_2_S_2_M_2_ without LHCB5 (see Figure 2B). Megacomplexes are averaged projections of 1 925 (C), 2 241 (D), and 2 305 (E) particles. (F) Isolated PSII particle from thylakoid membranes with “randomly” organized PSII as an average projection of 3 741 particles fitted by the model of PSII supercomplex C_2_S_2_M_2_ without LHCB5 (see Figure 2B). (G) PSII supercomplexes organized into 2D semi‐crystalline array as an average projection of 418 sub‐areas together with the fitted model of PSII C_2_S_2_M_2_ supercomplexes (see Figure 2B). Projection maps of PSII supercomplexes show core complexes in green, S trimers in magenta, M trimers in blue, LHCB4 in yellow, and LHCB6 in dark orange color.

Thylakoid membranes from both WT and the *lhcb5* mutant show regions where PSII are localized “randomly” as well as regions with ordered PSII arrays (Figure 3A, B). The area occupied by arrays in *lhcb5* mutant thylakoids is mostly below 10% (Figure S5), which agrees with what is typically seen in WT thylakoids (7–8%; Kouřil et al., 2013). Similarly to WT, these semi‐crystalline arrays in the *lhcb5* mutant can be nicely fitted using C_2_S_2_M_2_ type of PSII supercomplexes (Figure 3G). The 2D PSII array in the *lhcb5* mutant is characterized by lattice cell parameters a(nm) x b(nm): (27.0 ± 0.3) x (19.0 ± 0.2) and cell area of 509 ± 7 nm^2^, which are similar to PSII array parameters in WT (Kouřil et al., 2013).

**FIGURE 4:**
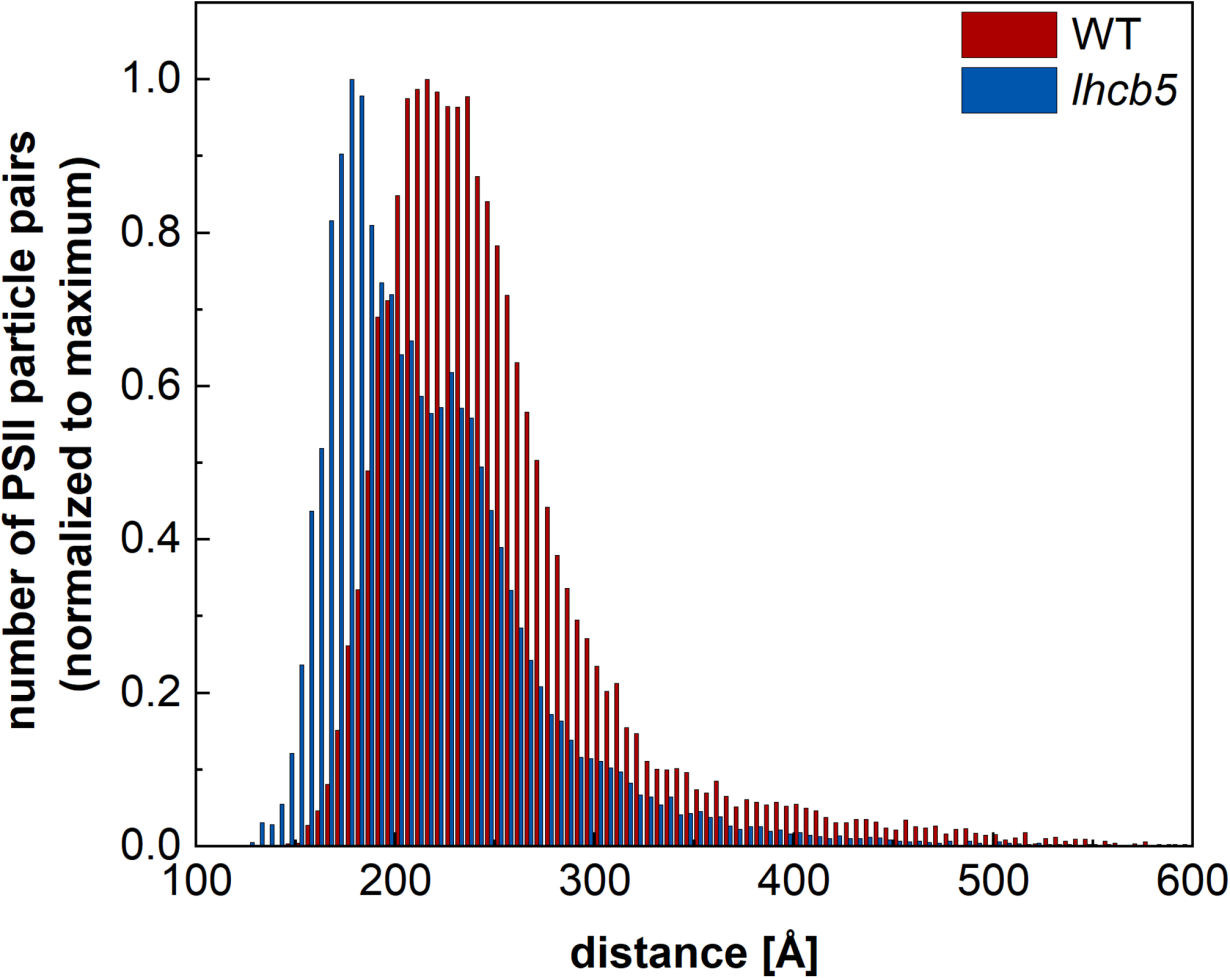
**Distribution of mutual distances between neighboring photosystem II particles in thylakoid membranes of Arabidopsis thaliana WT and the lhcb5 mutant**. The distances between two closest neighboring PSII supercomplexes were analyzed using EM. Histograms are normalized to the maximum.

To characterize the regions of thylakoid membranes with a seemingly “random” distribution of PSII complexes, we have determined the distribution of minimum distances between neighbouring PSII particles. The analysis revealed that the minimum distance between PSII particles in the membranes of WT was mostly around 222 Å, but surprisingly, the distribution for the *lhcb5* mutant appeared to have two local maxima (Figure 4). One (~ 227 Å) corresponded roughly to minimal distances between PSII in WT, but the second one – the global maximum – was much lower (~ 178 Å) and indicated that PSII particles in *lhcb5* are localized closer to each other (Figure 4).

To identify whether there are any specific interactions between PSII supercomplexes that would explain the tighter packing of PSIIs in the *lhcb5* mutant, we performed image analysis of all possible associations of two neighbouring PSIIs in the “random” regions of thylakoid membranes. The analysis revealed three specific associations of neighbouring PSII supercomplexes, characterized by mutual distances 176, 195 and 198 Å (Figure 3C‐E), which fall into the global maximum of PSII distance distribution in *lhcb5* (Figure 4). No megacomplexes with mutual PSII distances 220–230 Å (local maximum of PSII distance distribution; Figure 4) were found in *lhcb5* by the image analysis (Figure 3A‐G), suggesting that at these distances, PSII have only random orientation.

The result of the image analysis of thylakoid membranes answered our question about the megacomplexes in the *lhcb5* mutant – they indeed are present, but their stability is very low as they were not detected by CN‐PAGE (Figure 2A). What is interesting is that all the detected PSII megacomplexes in the *lhcb5* mutant were formed by parallel associations of PSII supercomplexes (Figure 3C‐E). Megacomplexes with non‐parallel associations, which were previously observed in WT thylakoids (Nosek et al., 2017), were completely absent. These findings suggest that minor antenna LHCB5 is important for the formation of non‐parallel associations of PSII megacomplexes and also for the overall stability of PSII megacomplexes.

In conclusion, this study provides the first structural characterization of the separated C_2_S_2_M_2_ PSII supercomplex in plants lacking LHCB5. Our findings reveal that the absence of LHCB5 is not compensated by any other light‐harvesting antenna protein, leaving its binding site in PSII vacant. This vacancy induces subtle distortions in the positioning of the S and M trimers, as well as LHCB6, within the supercomplex. These structural modifications weaken binding interactions, potentially contributing to the reduced stability of the PSII supercomplex compared to the wild type.

Not only the instability of C_2_S_2_M_(2)_ supercomplexes but also the absence of LHCB5 itself contribute to the observed instability of PSII megacomplexes and their absence in CN‐PAGE. Despite this, specific PSII megacomplexes, characterized by parallel‐associated PSII supercomplexes, were detected directly in the thylakoid membranes of the *lhcb5* mutant. This finding highlights the crucial role of LHCB5 in PSII organization within the thylakoid membrane, particularly in the formation of PSII megacomplexes containing non‐parallel‐associated PSII supercomplexes, which were absent in the *lhcb5* mutant.

The presence of C_2_S_2_M_(2)_ PSII supercomplexes distinguishes the *lhcb5* mutant from the mutants lacking the other minor LHCs, LHCB4 or LHCB6. This suggests that, unlike *lhcb4* and *lhcb6* mutants, the *lhcb5* mutant retains photosynthetic characteristics similar to wild‐type plants, possibly due to the preserved C_2_S_2_M_2_ supercomplex.

## AUTHOR CONTRIBUTIONS

T.V., P.I., R.K., and I.I. planned and designed the research, all authors performed experiments and analyzed the data. T.V., I.I., R.K. and P.I. wrote the manuscript with input from all authors, and all authors revised and approved it.

## FUNDING INFORMATION

This work was supported by the Czech Science Foundation (project no. 21‐05497S), grant No. IGA_PrF_2024_030 of Palacký University, and from the project TowArds Next GENeration Crops, reg. No. CZ.02.01.01/00/22_008/0004581 of the ERDF Programme Johannes Amos Comenius. We acknowledge the CEITEC Proteomics Core Facility of CIISB, Instruct‐CZ Centre, supported by MEYS CR (LM2023042, e‐INFRA CZ (ID: 90254)).

## CONFLICT OF INTEREST STATEMENT

The authors declare that they have no conflicts of interest with the contents of this article.

## Supporting information


**Appendix S1:** Supporting Information

## Data Availability

Datasets were deposited into a publicly accessible repository via Zenodo under DOI: 10.5281/zenodo.13819747. Mass spectrometry proteomics data were deposited to the ProteomeXchange Consortium via the PRIDE (Perez‐Riverol et al., 2019) partner repository under dataset identifier PXD052662.
