# Supplementary material for: Photosystem II supercomplexes lacking light‐harvesting antenna protein LHCB5 and their organization in the thylakoid membrane
Source: Physiol Plant. 2025 Mar 24;177(2):e70167. doi: 10.1111/ppl.70167 (PMC11932966; doi:10.1111/ppl.70167)
Supplement: Supplementary file 1 — Appendix S1: Supporting Information [file PPL-177-e70167-s001.pdf]

SUPPORTING INFORMATION

Manuscript title: Photosystem II supercomplexes and megacomplexes lacking light-harvesting antenna LHCB5

Authors: Tereza Vánská, Roman Kouřil, Monika Opatíková, Iva Ilíková, Rameez Arshad, Pavel Roudnický, Petr Ilík

Content of supporting information:

Supporting method 1
Supporting tables S1, S2
Supporting figures S1, S2, S3, S4, S5

Supporting method 1. Sample preparation for mass spectrometry analysis and data processing.

Samples of thylakoid membranes were each divided into 3 equal parts (creating technical replicates) from which proteins were extracted in SDT buffer (4% SDS, 0.1 M DTT, 0.1 M Tris/HCl, pH 7.6) in a thermomixer (Eppendorf ThermoMixer C, 15 min, 95 °C, 750 rpm). After that, samples were centrifuged (15 min, 20 000 *g*) and the supernatants (ca 50 µg of total protein) processed by filter-aided sample preparation (FASP) as described elsewhere (Wiśniewski et al., 2009) using 0.5 µg of trypsin (sequencing grade; Promega). Tryptic peptides were extracted info LC-MS vials by 2.5% formic acid (FA) in 50% ACN with addition of polyethylene glycol (20 000; final concentration 0.001%) (Stejskal et al., 2013) and concentrated in a SpeedVac concentrator (Thermo Fisher Scientific).

LC-MS/MS analyses of all peptide mixtures were done using nanoElute system (Bruker) connected to timsTOF Pro spectrometer (Bruker). Two column (trapping column: Acclaim™ PepMap™ 100 C18, dimensions 300 µm ID, 5 mm long, 5 µm particles, Thermo Fisher Scientific; separation column: Aurora C18, 75 µm ID, 250 mm long, 1.6 µm particles, Ion Opticks) mode was used on nanoElute system with default equilibration conditions (trap column: 10 volumes at 217.5 bars; separation column: 4 column volumes at 800 bars). Sample loading was done using 3 pickup volumes +2 µl at 100 bars. Trapped peptides were eluted by 90 min linear gradient program (flow rate 300 nl min^{-1}, 3-30% of mobile phase B; mobile phase A: 0.1% FA in water; mobile

phase B: 0.1% FA in acetonitrile) followed by system wash step at 80% mobile phase B. The analytical column was placed inside the Column Toaster (40 °C; Bruker) and its emitter side was installed into CaptiveSpray ion source (Bruker).

MSn data were acquired in m/z range of 100-1700 and 1/k0 range of 0.6-1.6 V s cm^{-2} using DDA-PASEF method acquiring 10 PASEF scans with scheduled target intensity of 20 000 and intensity threshold of 2 500. Active exclusion was set for 0.4 min with precursor reconsideration for 4× more intense precursors.

For DDA data evaluation, we used MaxQuant software (v2.0.3.0) (Cox & Mann, 2008) with inbuild Andromeda search engine (Cox et al., 2011). Search was done against protein databases of *Arabidopsis thaliana* (ftp://ftp.uniprot.org/pub/databases/uniprot/current_release/knowledgebase/reference_proteomes/Eukaryota/UP000006548/UP000006548_3702.fasta.gz; version 2021/11, number of protein sequences: 27 469) and cRAP contaminants (112 sequences, version from 2018/11, downloaded from http://www.thegpm.org/crap). Modifications were set as follows for database search: oxidation (M), deamidation (N, Q), acetylation (Protein N-term) as variable modifications and carbamidomethylation (C) as fixed modification. Enzyme specificity was tryptic/P with two permissible missed cleavages. Only peptides and proteins with false discovery rate threshold under 0.01 were considered. Match between runs was switched on with the default settings. Relative protein abundance was assessed using protein intensities calculated by MaxQuant. Intensities of reported proteins were further evaluated using software container environment (https://github.com/OmicsWorkflows/KNIME_docker_vnc; version 4.1.3a). Processing workflow is available upon request and it covers, in short, reverse hits and contaminant protein groups (cRAP) removal, protein group intensities log2 transformation and normalization (loessF).

Mass spectrometry proteomics data were deposited to the ProteomeXchange Consortium via PRIDE (Perez-Riverol et al., 2019) partner repository under dataset identifier PXD052662. Account details enabling the reviewers to access the data are as follows: Username: reviewer_pxd052662@ebi.ac.uk; Password: GjNwRs9blyvi.

Reference list

Cox, J. & Mann, M. (2008) MaxQuant enables high peptide identification rates, individualized p.p.b.-range mass accuracies and proteome-wide protein quantification. *Nature Biotechnology*, 26, 1367–1372

Cox, J., Neuhauser, N., Michalski, A., Scheltema, R.A., Olsen, J. V. & Mann, M. (2011) Andromeda: A peptide search engine integrated into the MaxQuant environment. *Journal of Proteome Research*, 10, 1794–1805

Perez-Riverol, Y., Csordas, A., Bai, J., Bernal-Llinares, M., Hewapathirana, S., Kundu, D.J., Inuganti, A., Griss, J., Mayer, G., Eisenacher, M., Pérez, E., Uszkoreit, J., Pfeuffer, J., Sachsenberg, T., Yilmaz, Ş., Tiwary, S., Cox, J., Audain, E., Walzer, M., et al. (2019) The PRIDE database and related tools and resources in 2019: Improving support for quantification data. *Nucleic Acids Research*, 47, D442–D450

Stejskal, K., Potěšil, D. & Zdráhal, Z. (2013) Suppresion of Peptide Sample Losses in Autosampler Vials. *Journal of Proteome Research*, 12, 3057–3062

Wiśniewski, J.R., Zougman, A., Nagaraj, N. & Mann, M. (2009) Universal sample preparation method for proteome analysis. *Nature Methods*, 6, 359–362

Supporting tables

Table S1. Physiological parameters of Arabidopsis WT and *lhcb5* mutant plants.

	**WT**	***lhcb5***
Fresh weight [g]	1.2 ± 0.1	1.3 ± 0.3
Chlorophyll a+b [µg cm^{-2}]	11.21 ± 0.51	10.17 ± 1.21
Chlorophyll a/b	3.02 ± 0.07	3.00 ± 0.11
Carotenoids [µg cm^{-2}]	1.89 ± 0.08 *	1.69 ± 0.03 *
F_V/F_M	0.80 ± 0.01 *	0.78 ± 0.01 *
F_0 [r.u.]	1.00 ± 0.08	0.97 ± 0.06
F_M [r.u.]	1.00 ± 0.05 *	0.88 ± 0.04 *

Presented values are means ± SD. n = 6 for fresh weight, 4 for Chl a+b, Chl a/b, Car, 14 for F_V/F_M, F_0, and F_M in WT, and 12 for F_V/F_M, F_0, and F_M in *lhcb5* mutant. The F_0 and F_M parameters are normalized to the WT values.
WT and *lhcb5* data were tested for significant difference. The data that passed normality and equal variance tests were compared by Student t-test, the ones that failed the normality or equal variance tests were compared by Mann-Whitney rank sum test. Data that are statistically significantly different are marked with an asterisk (α ≤ 0.05).

Table S2. Densities of the PSII supercomplex bands in the *lhcb5* mutant plants related to their counterparts in WT plants.

PSII supercomplex	relative density
$C_2S_2M_2$	0.60 ± 0.07
C_2S_2M	0.74 ± 0.13
C_2S_2/C_2SM	1.35 ± 0.08

Presented values are means ± SD, n = 3. Evaluated bands represent PSII supercomplexes consisting of PSII core complex (C), strongly bound LHCII trimer (S), and moderately bound LHCII trimer (M). The bands were separated using CN-PAGE.

Supporting figures

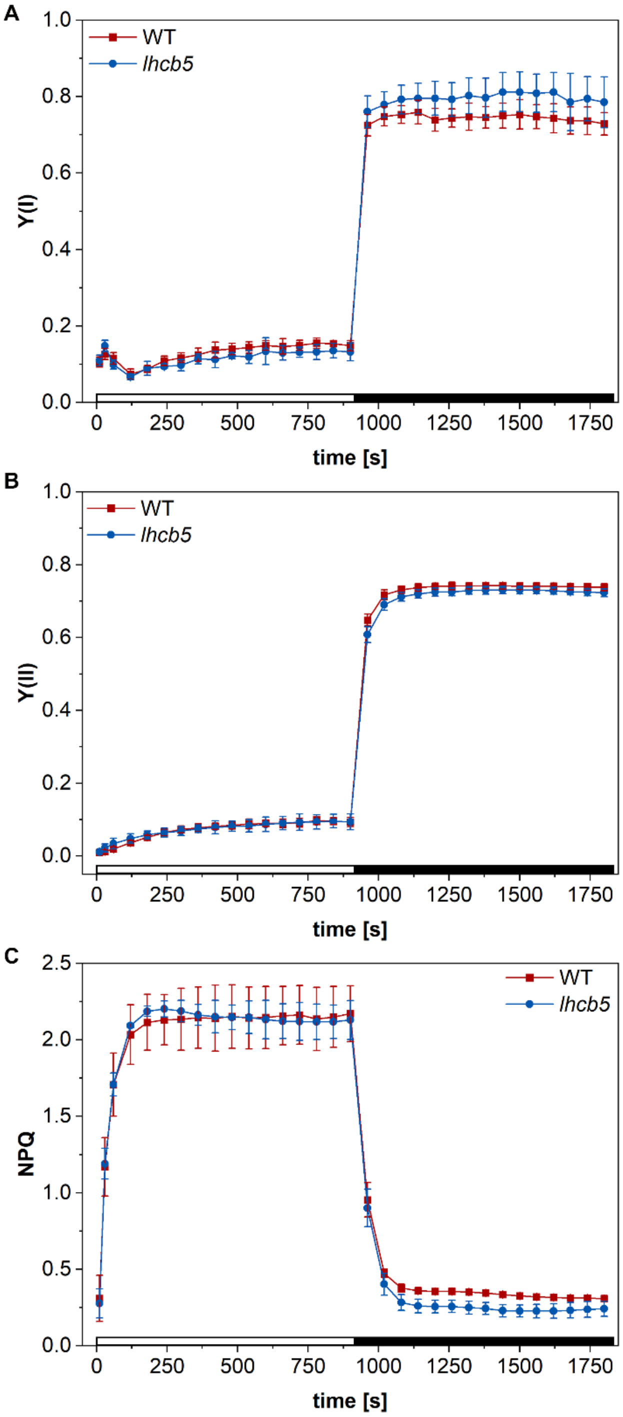

Figure S1. Selected photosystem I- and II-related parameters during light and dark period.
(A) Quantum yield of photochemistry of PSI - Y(I).
(B) Quantum yield of photochemistry of PSII - Y(II). F_V/F_M ratios for dark-adapted samples representing the maximum quantum yield of PSII photochemistry were 0.839 ± 0.007 and 0.814 ± 0.001 for for WT and *lhcb5* mutant, respectively.
(C) Non-photochemical quenching – NPQ.
Parameters were measured during actinic light exposure (marked with white bar, 800 μmol photons m^{-2} s^{-1}) and dark relaxation (marked with black bar) using saturating light pulses (300 ms, 10 000 μmol photons m^{-2} s^{-1}) in WT and *lhcb5* mutant plants. Results

represent mean values ± SD, n = 4. Plants were dark-adapted for 30 min before the measurement.

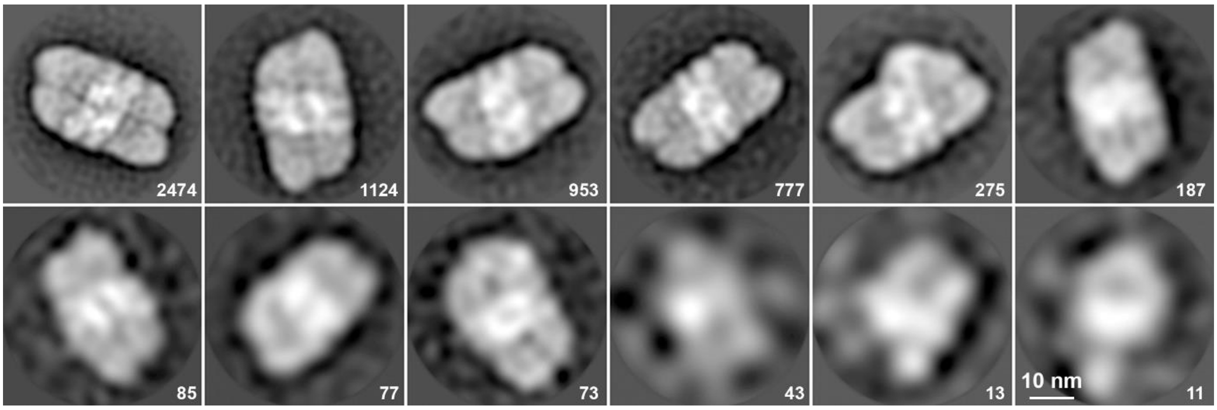

Figure S2. Single-particle image analysis and classification of protein complexes from CN−PAGE $C_2S_2M_2$ band from the Arabidopsis *lhcb5* mutant (Figure 2A).
Number of averaged projections in given classes are indicated.

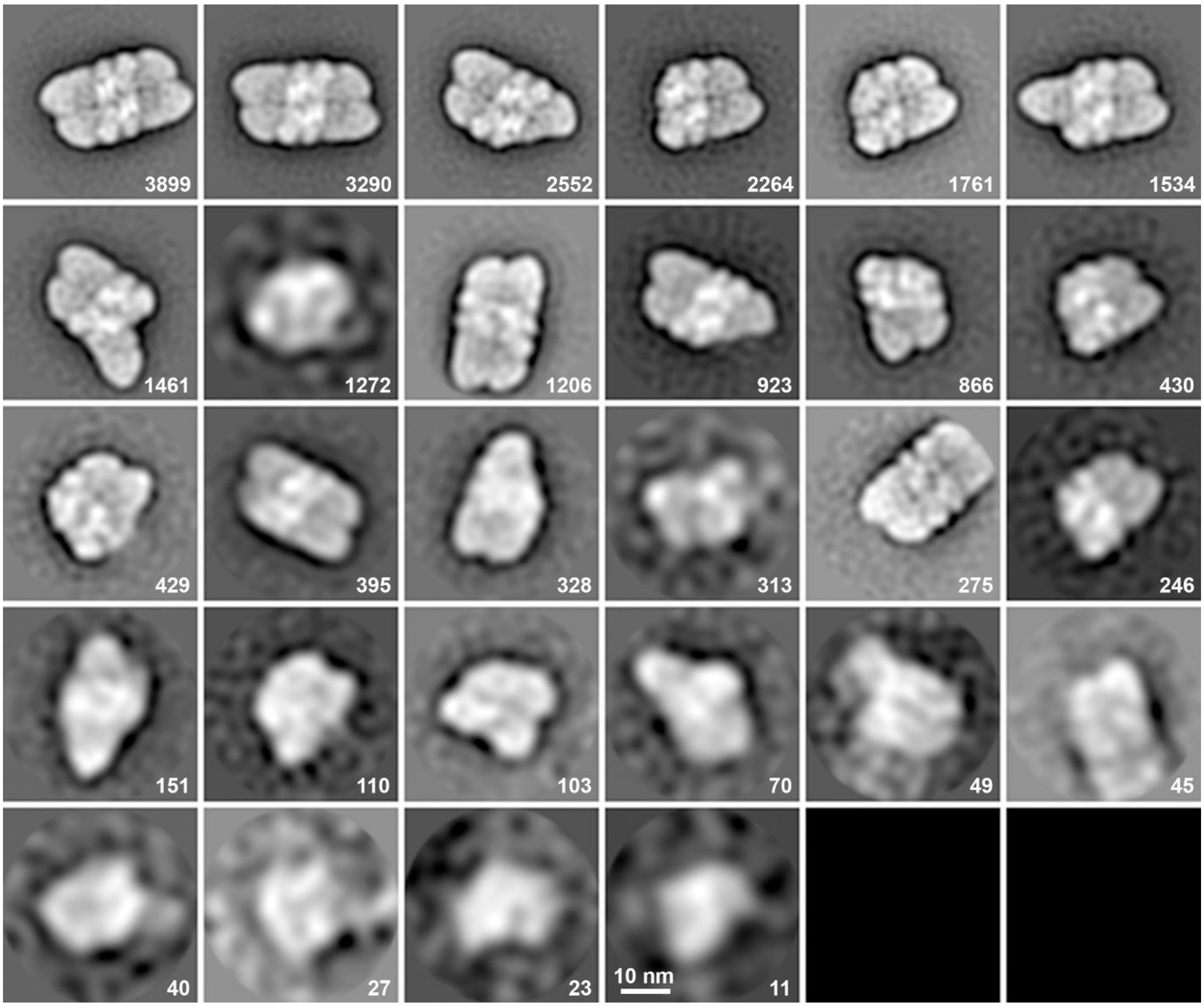

Figure S3. Single-particle image analysis and classification of protein complexes from CN−PAGE C_2S_2M band from the Arabidopsis *lhcb5* mutant (Figure 2A).
Number of averaged projections in given classes are indicated.

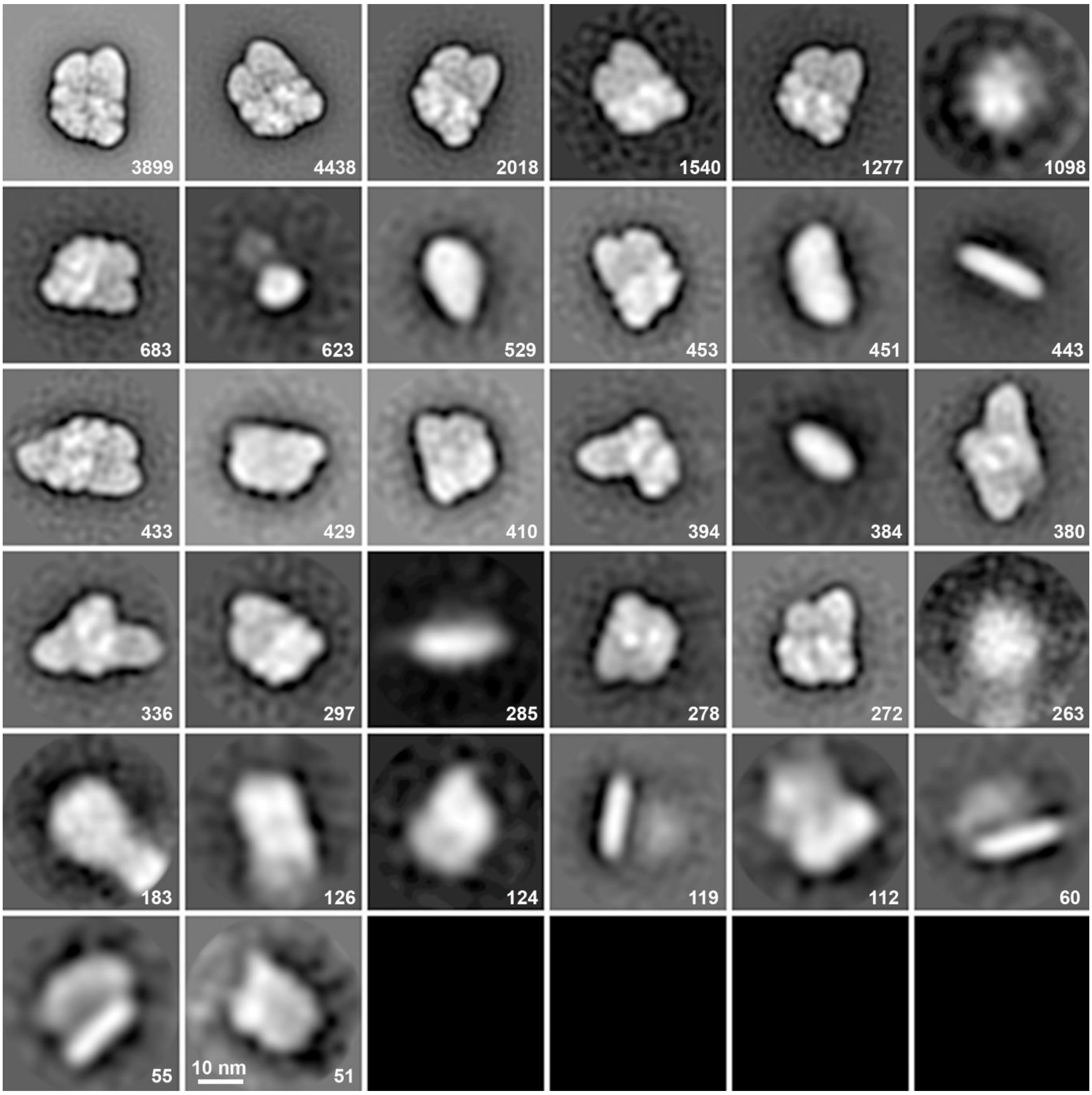

Figure S4. Single-particle image analysis and classification of protein complexes from CN−PAGE C_2SM band from the Arabidopsis *lhcb5* mutant (Figure 2A).
Number of averaged projections in given classes are indicated.

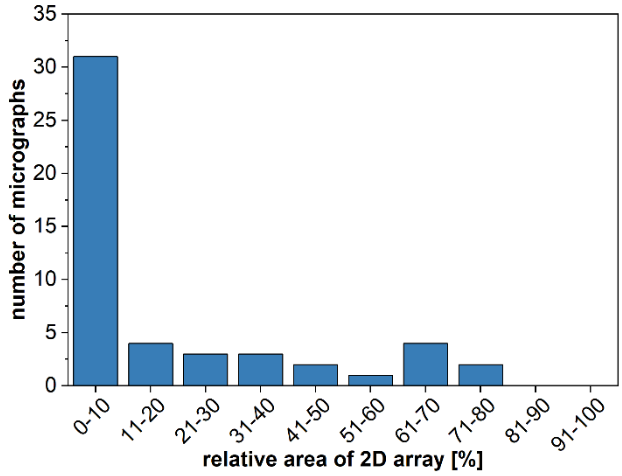

Figure S5. A histogram of the relative abundance of PSII semi-crystalline arrays in thylakoid membranes of Arabidopsis *lhcb5* mutant.

The bars represent the number of electron micrographs where the 2D arrays cover the indicated percentage of the membrane. The histogram was obtained by evaluation of 50 randomly selected images.
